# Adequately Adapted Insulin Secretion and Decreased Hepatic Insulin Extraction Cause Elevated Insulin Concentrations in Insulin Resistant Non-Diabetic Adrenal Incidentaloma Patients

**DOI:** 10.1371/journal.pone.0077326

**Published:** 2013-10-16

**Authors:** Christian-Heinz Anderwald, Andrea Tura, Alois Gessl, Anton Luger, Giovanni Pacini, Michael Krebs

**Affiliations:** 1 Division of Endocrinology and Metabolism, Department of Internal Medicine III, Medical University of Vienna, Vienna, Austria; 2 Metabolic Unit, Institute of Biomedical Engineering, National Research Council (ISIB-CNR), Padua, Italy; 3 Mariahilf Community Pharmacy, Arnoldstein, Austria; 4 Medical Direction, Specialized Hospital Complex Agathenhof, Micheldorf, Austria; National Health Research Institutes, Taiwan

## Abstract

**Background:**

Insulin-resistance is commonly found in adrenal incidentaloma (AI) patients. However, little is known about beta-cell secretion in AI, because comparisons are difficult, since beta–cell-function varies with altered insulin-sensitivity.

**Objectives:**

To retrospectively analyze beta–cell function in non-diabetic AI, compared to healthy controls (CON).

**Methods:**

AI (n=217, 34%males, 57±1years, body-mass-index:27.7±0.3kg/m^2^) and CON [n=25, 32%males, 56±1years, 26.7±0.8kg/m^2^] with comparable anthropometry (p≥0.31) underwent oral-glucose-tolerance-tests (OGTTs) with glucose, insulin, and C–peptide measurements. 1mg-dexamethasone-suppression-tests were performed in AI. AI were divided according to post–dexamethasone-suppression–test cortisol-thresholds of 1.8 and 5µg/dL into 3subgroups: pDexa<1.8µg/dL, pDexa1.8-5µg/dL and pDexa>5µg/dL. Using mathematical modeling, whole-body insulin-sensitivity [Clamp-like-Index (CLIX)], insulinogenic Index, Disposition Index, Adaptation Index, and hepatic insulin extraction were calculated.

**Results:**

CLIX was lower in AI combined (4.9±0.2mg·kg^-1^·min^-1^), pDexa<1.8µg/dL (4.9±0.3) and pDexa1.8-5µg/dL (4.7±0.3, p<0.04 vs.CON:6.7±0.4). Insulinogenic and Disposition Indexes were 35%–97% higher in AI and each subgroup (p<0.008 vs.CON), whereas C–peptide–derived Adaptation Index, compensating for insulin-resistance, was comparable between AI, subgroups, and CON. Mathematical estimation of insulin–derived (insulinogenic and Disposition) Indexes from associations to insulin-sensitivity in CON revealed that AI-subgroups had ~19%-32% higher insulin-secretion than expectable. These insulin-secretion-index differences negatively (r=-0.45, p<0.001) correlated with hepatic insulin extraction, which was 13-16% lower in AI and subgroups (p<0.003 vs.CON).

**Conclusions:**

AI-patients show insulin-resistance, but adequately adapted insulin secretion with higher insulin concentrations during an OGTT, because of decreased hepatic insulin extraction; this finding affects all AI-patients, regardless of dexamethasone-suppression-test outcome.

## Introduction

The advances in radiologic examinations have led to more frequent findings of nodules in the adrenal gland, also termed as adrenal incidentalomas (AI) [1]. Radiologic AI diagnosis has opened a new window of action to endocrinologists: On one hand, the clear absence of malignancy has to be proven by adequate imaging, and on the other hand, a check of this nodule’s endocrine activity is needed. However, the preponderant part of AI turns out to be benign and seems endocrine inactive [2]. 

Previous studies have suggested AI to be associated with the *Cardiometabolic Syndrome*: AI patients are insulin resistant with higher post-glucose-load plasma glucose concentrations and therefore increased prevalence of type-2 diabetes mellitus (T2DM) or glucose intolerance, as well as arterial hypertension and dyslipidaemia, all of which may contribute to the observed, greater cardiovascular risk [3-9]. Also osteoporosis is more frequent in AI patients [1,10]. Interestingly, some of these disturbances were at least in part reversible after surgical removal of AI [11]. However, despite those numerous studies on insulin resistance in AI, less attention has been paid to insulin secretion, another important predictor of T2DM development, as observed in T2DM–prone offspring of T2DM patients [12-14]. We hypothetisized that not only insulin sensitivity, but also insulin secretion might be altered in AI, since glucocorticoids could stimulate insulin release [15,16]. However, assessment of beta–cell function among individuals with different insulin sensitivity is not an easy task, because in non‑diabetic humans, insulin resistance is accompanied by a rise in insulin secretion, in order to compensate for the reduced action of insulin on responsive tissues [17,18]. 

It has been generally accepted for long that a repeated, fasting, post- dexamethasone-suppression-test (DST) cortisol level above 5µg/dL (pDexa5µg/dL) is considered abnormal and brings about diagnosis of overt Cushing syndrome [19], which goes along with signs and/or symptoms specific to overt cortisol excess, such as purple striae, easy bruising, proximal muscle weakness, and plethora [10,19]. More recently, however, a lower level for differentiation between patients with impaired and normal cortisol suppression has been proposed [10,19]. The utmost part of studies during the past decade defined this cut-off level at 1.8µg/dL for hypercortisolaemic subclinical Cushing syndrome, leading to higher incidence of T2DM, hypertension and osteoporosis, but not necessarily pronounced signs and/or symptoms of overt Cushing syndrome [10]. 

Thus, one of this study’s aims is to investigate metabolic parameters in non‑diabetic AI patients with sophisticated methods *in vivo*. We aimed to compare measures of whole–body insulin sensitivity, insulin secretion, glucose appearance and hepatic insulin extraction with those of healthy control subjects (CON). Moreover, beta-cell function was also related to insulin sensitivity to evaluate the ability of the beta-cell to adapt its secretion to changes in insulin resistance. In addition, according to the post-DST-cortisol thresholds of 1.8 and 5µg/dL mentioned above, an *in depth* analysis was performed by dividing AI patients into 3 subgroups: pDexa<1.8µg/dL, pDexa1.8-5µg/dL, and pDexa5µg/dL. Thus, our study design would also allow the solution to the question of clinicians whether the proven absence of even subclinical Cushing means unchanged insulin sensitivity and/or secretion, or should the mere knowledge of AI existence justify the assumption of altered metabolic parameters, regardless of the DST outcome.

## Materials and Methods

### Study participants

Patients with newly discovered incidentalomas by ultrasound, computer-, and/or magnetic resonance-tomography were admitted between 2000 and 2011 to the endocrine outpatients ward of our department. In total, 217 patients fulfilled the following inclusion criteria: (i) absence of diabetes mellitus and other known diseases such as in liver and/or kidney, or phaeochromocytomas (as far as extractable from the data), as well as (ii) performance of an oral glucose tolerance test (OGTT) with a routine baseline blood analysis including fasting serum cortisol and, (iii) a 1mg dexamethasone-suppression-test (DST) with measurement of serum cortisol at 8:00AM after dexamethasone consumption at 11:00PM the night before. The patients’ data were electronically composed using computer–assisted collection, by which all patients’ data and diagnoses in the described timeframe were included. The control group (CON) consisted of 25 healthy humans, who were age-, sex-, and body-mass-index-(BMI)-matched (Tab. 1), and did not take any regular medication known to affect insulin sensitivity, –secretion, hyperlipidaemia, and/or hypertension. The data composition as well as the study design and analyses were approved by the local ethics committee of the Vienna Medical University (#1970/2012). Because of the retrospective analysis, no consent was obtained from the patients in any form. The local ethics committee approved this procedure including the waiver of (another) (written) consent.

**Table 1:** Anthropometric measures, basal clinical laboratory values, results before and after the dexamethasone suppression test, OGTT results, such as fasting glucose and impaired glucose tolerance prevalence, OGTT AUCs, surrogates of insulin sensitivity, beta–cell secretion measures, hepatic insulin extraction, and fasting endogenous glucose production in controls (CON) and patients with adrenal incidentalomas (AI).

	**CON**	**AI**	**p (t-test)**
**Anthropometric characteristics and baseline lab**			
n	25	217	-
Sex (% males)	32%	34%	0.870
Age (years)	56±1	57±1	0.570
Body weight (kg)	78±3	79±1	0.683
Height (cm)	171±2	169±1	0.294
BMI (kg/m^2^)	26.7±0.8	27.7±0.3	0.310
Serum creatinine (mg/dL)	0.94±0.03	0.93±0.02	0.711
ASAT (U/L)	26±1	23±8	0.473
ALAT (U/L)	24±2	25±9	0.797
Total cholesterol (mg/dL)	230±6	220±3	0.255
LDL cholesterol (mg/dL)	144±5	134±2	0.157
HDL cholesterol (mg/dL)	65±3	60±1	0.112
Triglycerides (mg/dL)	106±9	139±5	**0.039**
Basal cortisol (µg/dL)	n.d.	16±0	-
Post dexamethasone test cortisol (µg/dL)	n.d.	2.1±0.2	-
Cortisol suppression by dexamethasone (%)	n.d.	87±1	-
Fasting glucose (mg/dL)	91±2	95±1	**0.034**
Impaired fasting glucose (%)	20%	26%	0.499
Glucose intolerance (%)	4%	24%	**0.020**
**OGTT area under the curves (AUCs)**			
AUC glucose (mg/dL·min)	13710±440	15835±231	**0.003**
AUC insulin (µU/mL·min)	4456±633	9502±376	**<0.001**
AUC C-peptide (ng/mL·min)	926±68	1178±27	**0.003**
**Surrogates of insulin sensitivity**			
QUICKI	0.426±0.007	0.407±0.004	0.105
ISI	6.3±0.4	3.9±0.2	**<0.001**
OGIS (mL/[min·m^2^])	435±11	379±4	**<0.001**
**Beta cell secretion measures**			
Basal insulin secretion rate per BSA (pmol/[min·m^2^])	95±11	115±4	0.124
Fasting beta-cell function (nmol/mmol)	0.15±0.01	0.17±0.01	0.242
IGI (0-60min) (pmol/mmol)	30±4	52±2	**<0.001**
IGI (0-120min) (pmol/mmol)	35±4	64±2	**<0.001**
Disposition Index	15±1	23±1	**<0.001**
Adaptation Index	160±11	152±3	0.420
WHOSH_CP	0.022±0.004	0.028±0.001	0.093
**Hepatic insulin extraction (%)**	67±2	52±1	**<0.001**
**Fasting EGP (mg·kg^-1^·min^-1^)**	1.11±0.09	1.29±0.04	0.108

Differences were analyzed by using the *Student*'s t–test: significant p–values are given in bold letters.

Abbreviations: ALAT, alanine aminotransaminase; ASAT, aspartate aminotransaminase; AUC, area under the curve; BMI, body mass index; BSA, body surface area; EGP, endogenous glucose production; HDL, high-density liproprotein; IGI, insulinogenic index; ISI, insulin sensitivity index; LDL, low-density liproprotein; OGIS, oral glucose insulin sensitivity; QUICKI, quantitative insulin sensitivity check index; WHOSH_CP, whole-OGTT-shape-index-C-peptide.

### Subgroup formation

In order to study the informative value of post-DST-cortisol, patients were divided into 3 subgroups: (i) pDexa<1.8µg/dL with post-DST-cortisol concentrations <1.8µg/dL, (ii) pDexa1.8-5µg/dL with post-DST-cortisol of 1.8-5µg/dL, and (iii) pDexa5µg/dL with post-DST-cortisol >5µg/dL (Tab. 2).

**Table 2:** Anthropometric measures, basal clinical laboratory values, results before and after the dexamethasone suppression test, OGTT results, such as fasting glucose and impaired glucose tolerance prevalence, OGTT AUCs, surrogates of insulin sensitivity, beta–cell secretion measures, hepatic insulin extraction, and fasting endogenous glucose production in controls (CON) and the 3 adrenal incidentaloma patients’ subgroups: pDexa<1.8µg/dL, post-DST-cortisol concentrations <1.8µg/dL; pDexa1.8-5µg/dL, 1.8-5µg/dL; and pDexa>5µg/dL, >5µg/dL.

	**CON**	**pDexa<1.8 µg/dL**	**pDexa1.8-5µg/dL**	**pDexa>5µg/dL**		**p (ANOVA)**
**Anthropometric characteristics and baseline lab**						
n	25	145	56	16	-	
Sex (% males)	32%	38%	27%	19%	0.264	
Age (years)	56±1	55±1 b	61±1 d	57±4	**0.002**	
Body weight (kg)	78±3	81±1	77±3	73±4	0.200	
Height (cm)	171±2	169±1 c	168±1	164±2 f	0.124	
BMI (kg/m^2^)	27±1	28±0	27±1	27±1	0.296	
Serum creatinine (mg/dL)	0.94±0.03	0.89±0.01 b c	0.98±0.04	1.04±0.12	**0.015**	
ASAT (U/L)	26±1	19±10	31±14	n.d.	**0.026**	
ALAT (U/L)	24±2	31±20	28±13	n.d.	0.451	
Total cholesterol (mg/dL)	230±6	221±3	218±6	217±13	0.640	
LDL cholesterol (mg/dL)	144±5	136±3	129±5	129±10	0.280	
HDL cholesterol (mg/dL)	65±3	58±1 a b	64±2	62±4	**0.019**	
Triglycerides (mg/dL)	106±9	146±7 a b	121±7	133±16	**0.036**	
Basal cortisol (µg/dL)	n.d.	15.3±0.4 c	16.9±0.8 e	20.8±1.5	**<0.001**	
Post dexamethasone test cortisol (µg/dL)	n.d.	1.1±0 b c	2.8±0.1 e	9.2±0.9	**<0.001**	
Cortisol suppression by dexamethasone (%)	n.d.	92±0 b c	82±1 e	53±4	**<0.001**	
Fasting glucose (mg/dL)	91±2	96±1 a	95±1	91±2	**0.045**	
Impaired fasting glucose (%)	20%	26%	27%	25%	0.924	
Glucose intolerance (%)	4%	24% a	29% d	13%	0.064	
**OGTT area under the curves (AUCs)**						
AUC glucose (mg/dL·min)	13710±440	15900±291 a	15912±436 d	14980±765	**0.017**	
AUC insulin (µU/mL·min)	4456±633	9668±486 a	9115±640 d	9360±1350 f	**<0.001**	
AUC C-peptide (ng/mL·min)	926±68	1151±32 a	1214±47 d	1297±145 f	**0.008**	
**Surrogates of insulin sensitivity**						
QUICKI	0.426±0.007	0.407±0.005	0.406±0.007	0.411±0.014	0.434	
ISI	6.3±0.4	4.0±0.3 a	3.8±0.3 d	4.1±0.7 f	**0.001**	
OGIS (mL/[min·m^2^])	435±11	373±5 a	389±8 d	400±16	**<0.001**	
**Beta cell secretion measures**						
Basal insulin secretion rate (pmol/[min·m^2^])	95±11	112±4	116±6	140±36 f	0.147	
Fasting beta-cell function (nmol/mmol)	0.15±0.01	0.17±0.01 c	0.18±0.01	0.22±0.06 f	0.064	
IGI (0-60min) (pmol/mmol)	30±4	51±3 a	52±4 d	55±8 f	**0.005**	
IGI (0-120min) (pmol/mmol)	35±4	65±3 a	62±4 d	69±10 f	**<0.001**	
Disposition Index	15±1	23±1 a	23±1 d	26±3 f	**<0.001**	
Adaptation Index	160±11	145±4 b c	161±6	187±21	**0.003**	
WHOSH_CP	0.022±0.004	0.028±0.001	0.027±0.002	0.034±0.006 f	0.215	
**Hepatic insulin extraction (%)**	67±2	51±1 a	53±2 d	54±5 f	**<0.001**	
**Fasting EGP (mg·kg^-1^·min^-1^)**	1.11±0.09	1.30±0.04	1.27±0.07	1.25±0.16	0.425	

Differences were analyzed by using ANOVA with LSD *post hoc* testing: significant ANOVA p–values are given in bold letters; *post hoc* differences with **p<0.05** among the groups by letters as follows:

a, pDexa<1.8µg/dL vs. CON; b, pDexa<1.8µg/dL vs. pDexa1.8-5µg/dL; c, pDexa<1.8µg/dL vs. pDexa5µg/dL; d, pDexa1.8-5µg/dL vs. CON; e, pDexa1.8-5µg/dL vs. pDexa5µg/dL; f, pDexa5µg/dL vs. CON.

Abbreviations: ALAT, alanine aminotransaminase; ASAT, aspartate aminotransaminase; AUC, area under the curve; BMI, body mass index; BSA, body surface area; EGP, endogenous glucose production; HDL, high-density liproprotein; IGI, insulinogenic index; ISI, insulin sensitivity index; LDL, low-density liproprotein; OGIS, oral glucose insulin sensitivity; QUICKI, quantitative insulin sensitivity check index; WHOSH_CP, whole-OGTT-shape-index-C-peptide.

Oral glucose tolerance test (OGTT). Participants were instructed to arrive at our endocrine outpatients ward in the morning in fasting condition, meaning an at least 10-hour period without consumption of food or beverages except water. Blood was drawn after insertion of a catheter (Vasofix^®^; Braun, Melsungen, Germany) into one antecubital vein at fasting, and 60, 90, and 120min after drinking a solution consisting of 75g glucose (Gluco-Drink75^®^; Roche Diagnostics, Vienna, Austria) for determination of plasma glucose and subsequent analyses of plasma hormones [20-22]. Samples were centrifuged and then either frozen at -80°C or immediately transported to the lab for rapid analyses.

### Measurements

Parameters of clinical chemistry, including serum cortisol, as well as circulating concentrations of glucose, insulin, and C-peptide, were measured at the laboratory of the Clinical Division of Endocrinology and Metabolism and/or the Department of Medical and Chemical Laboratory Diagnostics (www.kimcl.at), as described [20-22].

### Calculations

Measures of insulin sensitivity, such as the Clamp-like (CLIX), the *Matsuda* (ISI) and the oral glucose insulin sensitivity (OGIS) indexes, QUICKI, and those of beta–cell function, such as the basal insulin secretion rate and the Insulinogenic Index (IGI) of 0‑60min and 0‑120min, were assessed as described in details elsewhere [12,21-27]. The product of insulin sensitivity with an index of post‑hepatic insulin appearance (sometimes termed Disposition Index) and that with C-peptide derived beta-cell function (sometimes termed Adaptation Index) provides figures of the capacity of the beta-cell to adapt its secretion to the changes in insulin resistance. A novel insulin secretion index derived from OGTT C-peptide concentrations, called WHole‑Ogtt‑SHape‑index‑C‑Peptide (WHOSH_CP), was determined as described elsewhere [28]. Areas under the curve (AUC) were calculated by using the trapezoidal rule. Hepatic insulin extraction (as percentage of the secreted hormone) was estimated as previously described [25]. In addition, for the calculation of endogenous glucose production (EGP), we exploited our recent findings that basal hepatic insulin sensitivity, which was calculated as 100 divided by EGP times fasting insulin secretion, equals ISI–HOMA, the inverse value of HOMA‑IR [27]; the rationale of this was confirmed in detail elsewhere [18,29]. Basal endogenous glucose production therefore equals 100 divided by ISI–HOMA times basal insulin secretion and is given in mg·kg^-1^·min^-1^. 

### Statistical analyses

All data are given as means±SEM. Before further analysis, the distribution of the variables was tested by visual examination for marked non-normality and/or the Kolmogorov-Smirnov test, yielding that every variable was normally distributed. Comparisons between two, or more than two groups, were done by using two-tailed unpaired Student’s t-tests, or ANOVA with *post hoc* least significant difference (LSD) tests, respectively. Linear methods were used for correlation analyses using *Pearson*'s correlation coefficient *r*, or if logarithmic, by *Spearman*’s method. Differences were considered statistically significant at p-values≤0.05. Statistical analyses were performed using SPSS^®^ (SPSS Inc., Chicago, IL) computer software.

## Results

### All AI patients combined (Tab. 1+Fig.1)

**Figure 1:**
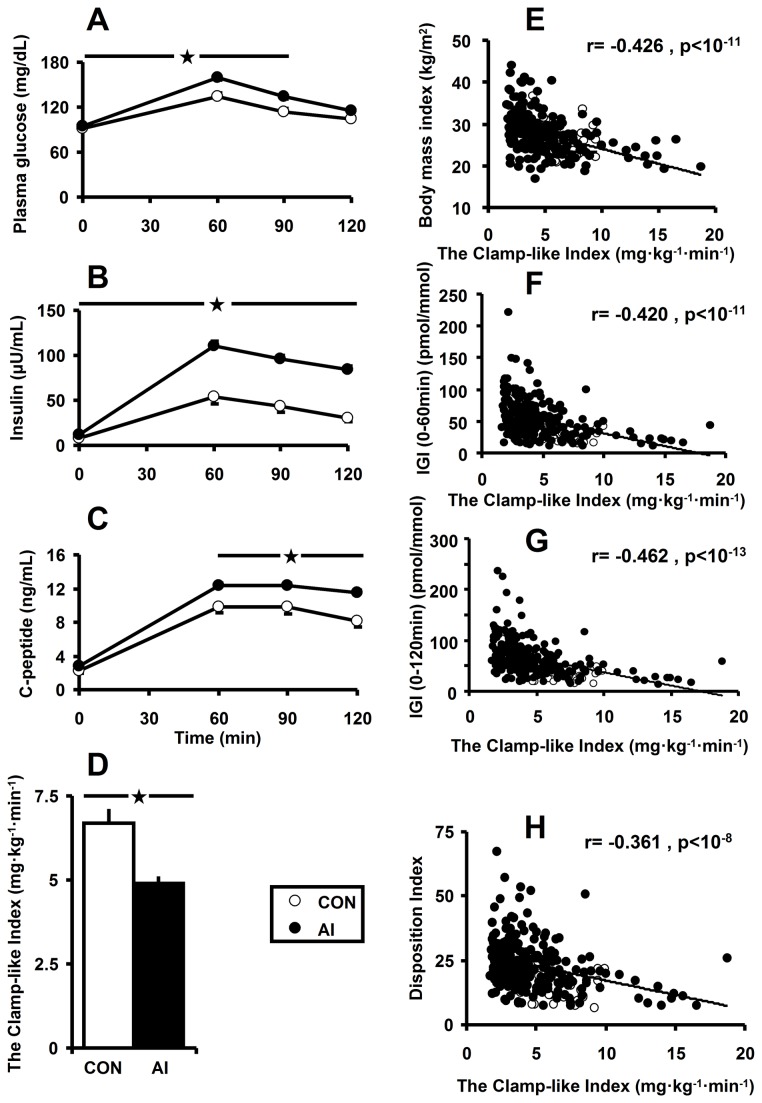
Circulating concentrations of glucose (A), insulin (B), C–peptide (C), and whole–body insulin–sensitivity by the Clamp-like Index (D), as well as *Pearson*’s product moment correlations between the Clamp-like Index on X–axis and on Y-axis body mass index (E), Insulinogenic Index (IGI, 0–60min; F), Insulinogenic Index (0–120min; G) and Disposition Index (H) in controls (CON, n=25, o) and adrenal incidentaloma patients (AI, n=217, ●). Differences were statistically analyzed by using *Student*'s t–test: *, p<0.05.

Anthropometric characteristics, such as age, BMI, and sex, as well as liver and kidney parameters (transaminases and creatinine) were not different between AI and CON (Tab. 1). Fasting glucose concentrations were slightly, but significantly, higher in AI by 4mg/dL, and glucose intolerance was six-fold higher in AI (each p<0.04). Serum concentrations of total, LDL- and HDL-cholesterol were comparable between both groups, while triglycerides were 31% higher in AI. AI showed higher OGTT glucose, insulin, and C-peptide concentrations (Figure 1A-C), which resulted in increased AUCs (Tab. 1). Basal (i.e. hepatic) insulin sensitivity, calculated from QUICKI, was unaffected, whereas whole–body insulin sensitivity was clearly reduced in AI, as displayed by CLIX (Figure 1D), ISI, or OGIS (Tab. 1), the latter reflecting glucose clearance (each p<0.001). Post‑hepatic insulin-related indices, IGI and the Disposition Index, were elevated in AI. On the other hand, those derived from C-peptide (fasting beta–cell function, the Adaptation Index and WHOSH_CP) were similar between AI and CON. This fact is also reflected by a 15% reduction in AI of hepatic insulin extraction, while fasting EGP was comparable. 

### The three AI subgroups

**Figure 2:**
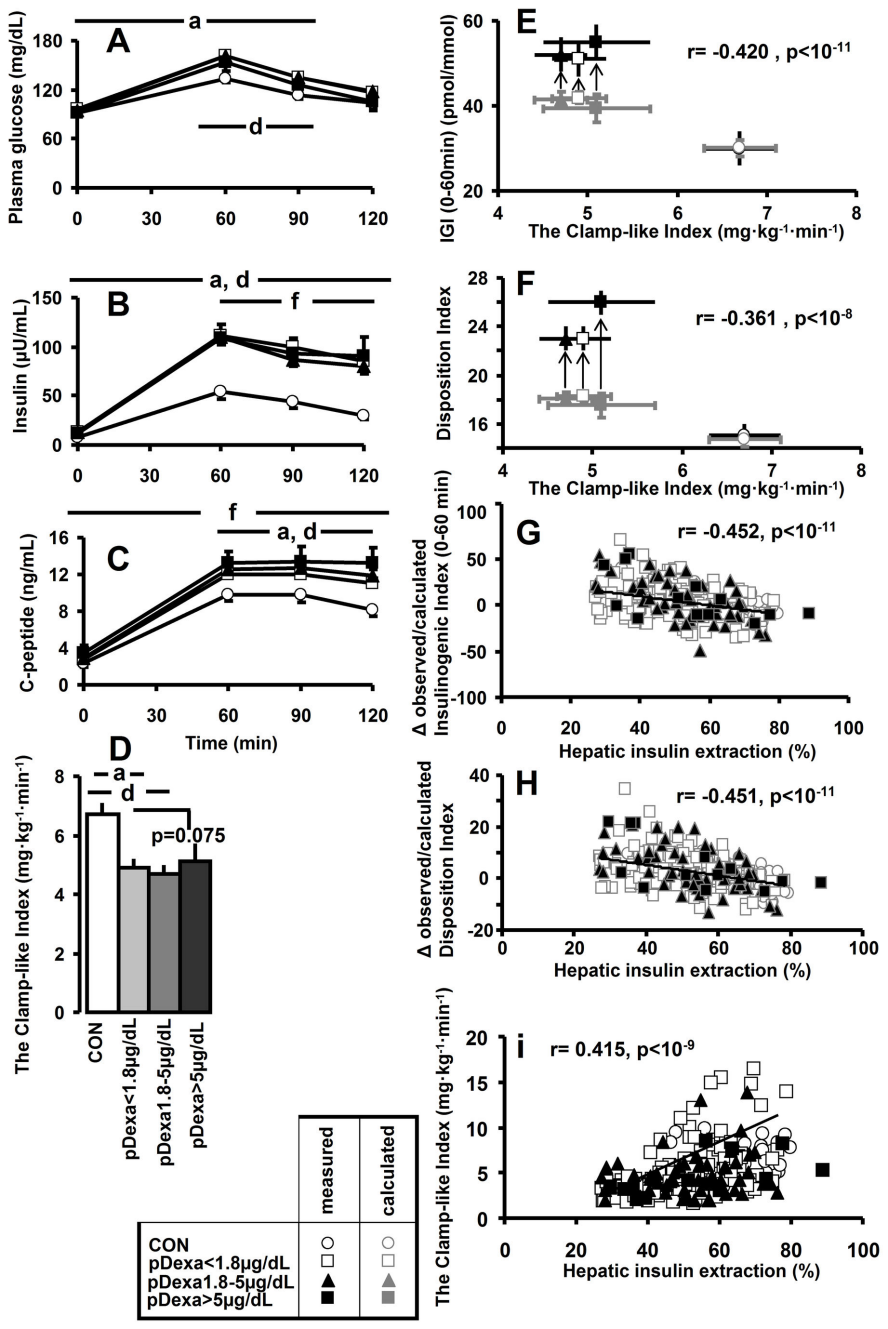
Circulating concentrations of glucose (A), insulin (B), and C–peptide (C), as well as whole–body insulin–sensitivity by the Clamp-like Index (D), as well as *Pearson*’s product moment correlations between Clamp-like Index and Insulinogenic Index (IGI, 0–60minutes; E) and the Disposition Index (F), as well as correlations between hepatic insulin extraction and Δ observed-calculated values of Insulinogenic Index (0–60minutes; G), Disposition Index (H) and Clamp-like Index (i) in the three AI subgroups [pDexa<1.8µg/dL (□, n=145), pDexa1.8-5µg/dL (▲, n=56), pDexa>5µg/dL (■, n=16)] and controls [CON, o, n=25). Symbols in panels **E** and **F** are given in black or in gray, if the values were obtained by measurement or calculation, respectively; arrows display the shift between expectable (i.e. calculated) and measured values. ANOVA with LSD *post* *hoc* test; *post* *hoc* differences with **p<0.05** among the groups are indicated by lowercase letters as follows: **a**, pDexa<1.8µg/dL vs. CON; **b**, pDexa<1.8µg/dL vs. pDexa1.8-5µg/dL; **c**, pDexa<1.8µg/dL vs. pDexa>5µg/dL; **d**, pDexa1.8-5µg/dL vs. CON; **e**, pDexa1.8-5µg/dL vs. pDexa>5µg/dL; **f**, pDexa>5µg/dL vs. CON.

Results are shown in Tab. 2 and Figure 2. Sex and BMI were similar among all 3 AI subgroups and CON, but age was higher in pDexa1.8-5µg/dL, when compared to pDexa<1.8µg/dL and CON (p<0.03). PDexa<1.8µg/dL showed lower high-density lipoprotein-(HDL)-cholesterol and higher triglyceride concentrations. Basal cortisol was highest in pDexa5µg/dL, when compared to both pDexa<1.8µg/dL and pDexa1.8-5µg/dL (p<0.001). Basal cortisol was different and rose, whereas suppression of fasting cortisol by DST fell among the subgroups (p<0.001). Fasting glucose concentrations were slightly higher by 5mg/dL in pDexa<1.8µg/dL than CON (p<0.05). PDexa<1.8µg/dL and pDexa1.8-5µg/dL showed 6- to 7-fold higher glucose intolerance. Circulating concentrations and AUCs of glucose, insulin, and C–peptide during OGTT (Figure 2A-C) were mostly higher in each AI subgroup, when compared to CON. Whole–body insulin sensitivity and glucose clearance, as determined by CLIX (Figure 2D), and OGIS (Tab. 2) were lower in pDexa<1.8µg/dL and pDexa1.8-5µg/dL, when compared to CON (each p<0.006), whereas also pDexa5µg/dL displayed lower insulin sensitivity, with regard to ISI (p<0.02). Fasting insulin secretion appeared higher in pDexa5µg/dL, and fasting beta–cell function in both pDexa<1.8µg/dL and pDexa5µg/dL. The Insulinogenic Index at 0-60min and 0-120min, as well as the Disposition Index were elevated in all 3 subgroups (each p<0.008 vs. CON), whereas no differences were found between all AI subgroups and CON with regard to the Adaptation Index. However, pDexa<1.8µg/dL had a slightly, but significantly, lower Adaptation Index than pDexa1.8-5µg/dL and pDexa5µg/dL (p<0.04). WHOSH_CP was 55% higher in pDexa5µg/dL. Again, hepatic insulin extraction was lower by 13-16% in each AI subgroup than in CON (p<0.004), while fasting EGP was comparable among all subgroups and CON.

### Correlation analyses (Figure 1E-H+Fig.2E-i)

In all AI patients combined and CON, there were strong inverse relationships between CLIX and BMI (r=-0.426, p<0.001), IGI at 0-60min (r=-0.420, p<0.001), and 0-120min (r=-0.462, p<0.001), as well as Disposition Index (r=-0.361, p<0.001) (Figure 1E-H), but not the Adaptation Index. Fasting beta–cell function was positively correlated with basal (r=0.147, p<0.04) and post-(r=0.162, p<0.02)-DST-cortisol concentrations. Post–DST–cortisol was positively related to Adaptation Index (r=0.193, p<0.005). No significant associations between basal and post–DST–cortisol were found with regard to insulin sensitivity parameters, such as CLIX, ISI, OGIS, and QUICKI. CLIX correlated positively with circulating concentrations of HDL-cholesterol (r=0.196, p<0.003) and negatively with those of triglycerides (r=-0.288, p<0.001).

In order to provide a more subtle assessment of beta-cell function in AI subgroups, we calculated the associations of CLIX with Insulinogenic Index (IGI, 0–60min) and Disposition Index (DI) in CON, which significantly (each p<0.04) follow the formulas: IGI = 83.0 - 28.7 x log_e_(CLIX) and DI = 30.4 - 8.5 x log_e_(CLIX). When applying these formulas to AI subgroups, we obtained lower calculated than measured results of both Insulinogenic and Disposition Indexes, as depicted as gray symbols in Figure 2E+F. The differences between observed and calculated values of both Insulinogenic Index (r=-0.452, p<0.001) and the Disposition Index (r=-0.451, p<0.001) were very closely negatively associated with hepatic insulin extraction (Figure 2G+H), which was positively related to measures of insulin sensitivity, such as CLIX (r=0.415, p<0.001) (Figure 2i), OGIS (r=0.645, p<0.001), ISI (r=0.649, p<0.001), and QUICKI (r= 0.516, p<0.001).

## Discussion

This retrospective study was undertaken in a large cohort of more than 200 non-diabetic patients with adrenal incidentaloma diagnosis to investigate whole–body insulin sensitivity and its relation to insulin secretion, as well as hepatic insulin extraction and endogenous glucose production, by applying advanced index calculation and sophisticated methods to OGTT data. AI patients underwent a 1mg dexamethasone-suppression-test for diagnosis of (subclinical) Cushing syndrome. 

This study’s major results in non-diabetic AI are: (i) reduced insulin sensitivity with higher circulating concentrations of glucose, insulin, and C-peptide during OGTT, (ii) increased insulin secretion and beta-cell function, (iii) diminished hepatic insulin extraction, (iv) negative association of insulin sensitivity with peripheral insulinaemia, but (v) C-peptide-derived Adaptation Index comparable to that of CON that shows a normal capacity of compensating for insulin resistance by augmenting insulin release. 

By dividing AI according to post‑dexamethasone-suppression-test cortisol threshold levels of 1.8 and 5µg/dL, three subgroups were created: pDexa<1.8µg/dL, pDexa1.8-5µg/dL, and pDexa5µg/dL. In these subgroups, we found: (vi) insulin resistance clearly present in pDexa<1.8µg/dL and pDexa1.8-5µg/dL; (vii) beta-cell function elevated, hepatic insulin extraction reduced, and the Adaptation Index still comparable to that of CON; (viii) that the adjustment of insulin–derived beta–cell indexes from relationships in CON measured values in AI subgroups to be higher than expected, and (ix) the differences between measured and calculated levels tightly and negatively correlated with hepatic insulin extraction; finally, (x) that post-DST-cortisol concentrations were positively associated with fasting beta-cell function and the Adaptation Index.

### Whole-body insulin sensitivity

OGTT–derived indexes of whole-body insulin sensitivity showed a clear reduction in AI, the subgroups pDexa<1.8µg/dL and pDexa1.8-5µg/dL, and a borderline decrease in pDexa5µg/dL. Surrogate indexes however did not agree: in fact, according to ISI, also the latter group exhibited a significant insulin resistance. This impairment, which was frequently shown in AI previously [3,5-9], is the core of the *Cardiometabolic Syndrome* and contributes to higher cardiovascular disease risk and complications proper of these patients [4]. Of note, the degree of insulin resistance observed in this study in non‑diabetic AI can be seen as impressive: healthy, non‑diabetic subjects with a BMI on average in the overweight, but not obese range, are expected to display an insulin sensitivity of ~7mg·kg^-1^·min^-1^ from the clamp–test, as others and ourselves have previously shown [22]. This expected result was seen only in the healthy overweight controls, but not the matching AI, whose insulin sensitivity was on average even below the threshold of pronounced insulin resistance of <5mg·kg^-1^·min^-1^ [22]. Our finding would correspond to an obesity‑like insulin sensitivity [22], meaning that all our AI patients should be considered at risk for disease and mortality, as if they were several kilograms heavier. This interesting outcome could not only contribute to explain their higher cardiovascular risk [4], but – to our surprise – affects all AI patients, also the pDexa<1.8µg/dL, and should be therefore spread over any AI patient, regardless of the DST outcome.

Moreover, the possible presence of liver insulin resistance can be assessed by QUICKI and EGP [29,30], which were comparable to those of CON in all AI patients combined and in every subgroup. This means that in fasting condition, these patients behave normally in terms of insulin sensitivity, but show their impairment only in dynamic conditions after a glucose load. Since, in general, non–diabetic subjects still have unaltered fasting EGP, regardless of presence or absence of insulin resistance [30,31], this study confirms the non-diabetic state of our patients.

### Insulin secretion

Insulin secretion in general rises since the very beginning of the appearance of the condition of insulin resistance, aiming for compensation of reduced action to insulin [32]. Insulin secretion in AI patients is comparable to that of healthy control subjects, i.e. adequately adapted to the relative degree of insulin resistance, as shown by the C–peptide-derived Adaptation Index, not different from CON in every subgroup. On the other hand, the indexes based on post-hepatic insulin levels were higher than CON and tightly and negatively related to hepatic insulin extraction. From this, it follows that higher OGTT insulin concentrations observed in AI were due to a higher post-hepatic insulin release associated to a lower extraction, proper of insulin–resistant states [33,34]. The reasons for this lower insulin extraction by the liver are still obscure: it may be the interplay of insulin resistance [33,34] and/or glucocorticoid excess, which may also lead to this phenomenon [35].

Another, interesting finding of this study was the positive association of post-DST-cortisol to both fasting beta–cell function and the Adaptation Index. Of note, in healthy people, the dexamethasone-suppression-test examines the remaining cortisol release in the adrenal cortex following short-term suppression of hypothalamic ACTH secretion. Thus, the post-DST–cortisol level can be regarded as adrenal excess production, most likely autonomously. Exposure of beta–cells to glucocorticoids resulted in beta-cell expansion and enhanced insulin secretion, but also a blunted C–peptide secretion upon stimulation [36], which was found in previous studies of ourselves in glucocorticoid-treated, non‑diabetic, renal transplant patients [25], and healthy humans [16]. Interestingly, in this study, we did not only observe increased insulin secretion due to reduced extraction in AI, but also alterations in the OGTT C–peptide shape, which was borderline higher in AI, and increased in pDexa5µg/dL, as determined by the novel WHOSH_CP‑index [28]. Another evidence for a rather slight stimulation of insulin secretion by cortisol overproduction seems the weak, but significantly positive association of post-DST-cortisol concentrations with Adaptation Index during OGTT and beta-cell function at fasting.

Another issue that might be associated with AI is the more frequent co–appearance of phaeochromocytomas. As far as possible by using the database, we have excluded all patients with phaeochromocytoma. In this context it is of note that phaeochromocytoma-derived catecholamines not only worsen insulin sensitivity, but also insulin secretion via alpha-2- adrenoceptors in beta-cells, which may contribute to diabetes mellitus development [37,38].

### Limitations

The major drawbacks of this study were on the one side the retrospective analyses with known disadvantages, and, on the other side, the measurement of metabolite and hormone concentrations at only two time-points within the first OGTT hour. However, none of the calculated parameters and indexes was greatly affected thereby, so that the main question seems to be solved. In a clinical setting with a high number of patients to be dealt with, less frequent OGTT blood sampling seems justified and unavoidable, owing to limited time slots in outpatients’ routine treatment.

## Conclusions

Patients with adrenal incidentalomas show insulin resistance, but adequately adapted insulin secretion with higher insulin concentrations during a glucose challenge, due to a decreased hepatic insulin extraction. These findings affect all AI patients, regardless of the outcome of the dexamethasone-suppression-test so that AI diagnosis seems to bring about high likelihood of metabolic alterations involved in the *Cardiometabolic syndrome*.
